# Obstetric and Gynecologic Nursing

**Published:** 1901-05

**Authors:** 


Obstetric and Gynecologic Nursing. By E. P. Davis, A. M., M. D., Professor of Obstetrics in Jefferson Medical College and Philadelphia Polyclinic. 12mo, volume of 402 pages, fully illustrated. Philadelphia and London: W. B. Saunders & Co., 1901. Price, $1.75 net.

This excellent little volume is intended primarily for the use of nurses, but it contains many details as to the management of obstetric and gynecologic cases, which the average physician will do well to note. Lack of attention to the "little things" in the management of these cases is responsible for many a practitioner's failure to get good results.

The illustrations in this book deserve especial mention, being unusually good and sufficiently numerous to well illustrate the text.

